# A Murine Model of Food Allergy by Epicutaneous Adjuvant-Free Allergen Sensitization Followed by Oral Allergen Challenge Combined with Aspirin for Enhanced Detection of Hypersensitivity Manifestations and Immunotherapy Monitoring

**DOI:** 10.3390/nu15030757

**Published:** 2023-02-02

**Authors:** Keiko Kameda, Etsuhisa Takahashi, Takashi Kimoto, Ryoko Morita, Satoko Sakai, Mizuho Nagao, Takao Fujisawa, Hiroshi Kido

**Affiliations:** 1 Division of Enzyme Chemistry, Institute for Enzyme Research, Tokushima University, Tokushima 770-8503, Japan; 2 Allergy Center and Institute for Clinical Research, Mie National Hospital, Tsu 514-0125, Japan

**Keywords:** food allergy, murine model, aspirin, skin sensitization, symptom score, enhanced detection, oral allergen challenge, chicken’s egg OVM allergy, cow’s milk casein allergy

## Abstract

Food allergy is one of the major existing health problems, but no effective treatment is available. In the current work, a murine model that closely mimics pathogenesis of human food allergy and its quantifiable diagnostic parameter design, even for mild hypersensitivity reactions, were established. BALB/c mice were epicutaneously sensitized with 1 mg chicken egg ovomucoid (OVM) or cow’s milk casein, free of adjuvants, five times a week for two consecutive weeks. Eleven days later, allergen-specific IgG1 and IgE in serum were measured by ELISA. On day 25, 20 mg OVM or 12 mg α-casein was administered orally, and allergic reactions such as the fall in rectal temperature, symptom scores during 90–120 min, serum mast cell protease-1 and cytokine levels were monitored. The detection of mild allergic reactions due to adjuvant-free allergen sensitization and oral allergen challenge routes was amplified by the combination of oral allergen and aspirin administration simultaneously or aspirin administration within 15–30 min before an allergen challenge. Quantification of the maximum symptom score and the frequency of symptoms during the monitoring period improved evaluation accuracy of food allergy signals. Based on these results, efficacy of casein oral immunotherapy for cow’s milk allergies, which are generally difficult to detect, was monitored adequately.

## 1. Introduction

The prevalence of food allergy has continued to rise over the past several decades and currently affects 6% to 13% of the world population, making it an important global health problem [1]. Antigen avoidance, antigen elimination diets and symptomatic treatment with antihistamines and other medications are currently considered the standard treatments. Immunotherapy has been developed for food allergy, but there are still many issues to be addressed, such as more effective and shorter treatment methods and more effectively avoiding the risk of adverse reactions, sometimes including anaphylaxis [1]. For further analysis of the mechanism of food allergy onset and development of new treatment modalities, it is desirable to develop widely accepted standardized animal models that closely resemble the onset mechanisms of human food allergy [2], that is adjuvant-free allergen sensitization and adjuvant-free oral allergen challenge, as well as the design of sensitive evaluation methods.

Many research groups have recently confirmed the hypothesis that food allergy in infants and children is caused by epicutaneous allergen sensitization at the sites of skin inflammation, such as atopic dermatitis [3]. The hypothesis was also tested and confirmed by clinical studies of peanut allergy by Lack and coworkers [4] and for chicken egg allergy by our group [5]. Naturally occurring food allergy does not exist in rodents, and, for this reason, strong and non-natural sensitization with adjuvants has been used in most experimental food allergy rodent models, such as oral allergen administration combined with an adjuvant cholera toxin, or intraperitoneal allergen administration with alum [6]. However, the use of adjuvants may induce specific IgE responses against proteins that are usually not considered as allergenic [7,8]. Furthermore, allergen challenge experimental studies designed to evoke allergic reactions in mice also involve the use of non-natural allergen administration routes, such as intraperitoneal or intravenous administration, rather than the natural oral route via gastrointestinal immunity. Therefore, the results of such experiments are difficult to apply to the analysis of the pathogenesis of human food allergy and to improve human immunotherapy.

In this study, we aimed to establish mouse models treated with adjuvant-free allergen sensitization and adjuvant-free oral allergen challenge, and to design quantifiable and sensitive diagnostic parameters for the treatments of food allergy. Mice were epicutaneously sensitized with either of the two major common food allergens in infants: chicken egg ovomucoid (OVM) or cow’s milk casein [9,10]. The onset of food allergy was confirmed by the increase in levels of blood allergen-specific IgE (sIgE), IgG1 (sIgG1), mouse mast cell protease-1 (mMCPT-1) and cytokine levels. The severity of allergic reactions induced by oral allergen challenge was evaluated by the fall in rectal temperature and by modified evaluation methods of symptom score reported [11,12,13]. To amplify the detection of mild allergic symptoms following oral allergen challenge, we established the method of oral aspirin treatment, including precise treatment timing relative to allergens in the food allergy mouse models. Finally, we introduced casein immunotherapy monitoring data in adjuvant-free casein-sensitized cow’s milk allergy mouse models.

## 2. Materials and Methods

### 2.1. Mice

The experiment was conducted in 7-week-old BALB/c female mice purchased from Japan SLC, Inc. (Shizuoka, Japan). All mice were kept under specific pathogen-free conditions and controlled conditions of humidity (40 ± 5%) and temperature (25 ± 1 °C). All animals were treated according to the Guide for the Care and Use of Laboratory Animals (NIH Publication No. 85–23, 1996). This study was approved by the Ethics Committee of Animal Care and Experimentation of Tokushima University (#T29-93 and T2020-81).

### 2.2. Antigens and Reagents

OVM, the trypsin inhibitor from chicken egg whites, and casein from cow’s milk were purchased from Nacalai Tesque (Kyoto, Japan). Acetylsalicylic acid (aspirin: ASA), lysine acetylsalicylic acid (lysine-aspirin: L-ASA) and α-casein from cow’s milk were purchased from Sigma-Aldrich (St. Louis, MO, USA). Sodium dodecyl sulfate (SDS) and ethanol (EtOH) were purchased from FUJIFILM Wako Chemicals Corporation (Osaka, Japan). Mouse MCPT-1 (mMCPT-1) uncoated enzyme-linked immunosorbent assay (ELISA) kit was purchased from Invitrogen (Waltham, MA, USA).

### 2.3. Epicutaneous Sensitization

For epicutaneous sensitization, the shaved back skin was treated with 4% SDS in sterile distilled water in order to damage tight junction barrier integrity in the intraepidermal granule cell layer of the skin [14,15]. After SDS treatment for 10 min, 1 mg of OVM or casein in 100 µL of saline was applied to the skin. This process of allergen sensitization was conducted five times a week in the first 2 weeks of the study (from day 0 to day 11). Naïve mice served as negative controls.

### 2.4. Measurement of Allergen sIgE, sIgG1 and Mouse Mast Cell Protease-1

The titers of sIgE and sIgG1 were measured by ELISA using mouse serum collected on day 11 after the initial sensitization. In these assays, the 96-well NUNC-immunoplate (Thermo Fisher Scientific, Waltham, MA, USA) was coated with allergen (1 µg/well) in carbonate buffer (pH 9.6) for 2 h at 37 °C, then washed 3 times with 50 mM Tris-HCl (pH 8.0) containing 0.15 M NaCl and 0.05% Tween 20 (TBST). The plate was blocked with 1% BSA in 50 mM Tris-HCl (pH 8.0) containing 0.15 M NaCl (TBS) for 2 h at 37 °C, and then washed three times with TBST. To measure sIgE levels, sera were serially diluted from 1:20 to 1:20^12^ with 1% BSA in TBST, added to each well (100 µL/well) and stood overnight at 4 °C. To measure sIgG1 levels, sera were serially diluted from 1:200 to 1:200^19^ with TBST containing 1% BSA, added to each well (100 µL/well) overnight at 4 °C. After washing five times with TBST, the plate was incubated with HRP-conjugated rat anti-mouse IgE (heavy chain) secondary antibody (Invitrogen) (100 µL/well of 1:2000 dilution) or HRP conjugated goat anti-mouse IgG1 (BETHYL, Montgomery, TX, USA) (100 µL/well of 1:10,000 dilution) for 2 h at 37 °C. After washing six times with TBST, the plate was incubated with TMB peroxidase substrate (SeraCare, Milford, MA, USA), according to the instructions provided by the manufacturer, for 15 min at room temperature. The reaction was subsequently quenched with 1 M sulfuric acid (50 µL/well), and absorbance at 450 nm was measured with a SpectraMax ABS PLUS-SC-ACAD (Molecular Devices, San Jose, CA, USA). The endpoint titers were expressed as the last dilution ratio for which the OD450 value was twice greater than that of the plate blank.

mMCPT-1 levels in serum were measured by using an uncoated ELISA kit according to the protocol supplied by the manufacturer. Serum was collected before oral allergen challenge and 2–24 h after oral load.

### 2.5. Spleen Cell Culture and Cytokine Productions

At the end of the one-month oral allergen challenge, isolated splenocytes from OVM-sensitized mice or naïve mice without any treatment were incubated with complete RPMI medium with or without 1 mg OVM/4 × 10^6^ cells/700 μL/well for 3 days at 37 °C as previously described [16]. The concentrations of IL-4, IL-5, IL-6, IL-9, IL-13 and IFN-γ in the culture media supernatant were measured by LEGEND-plex^TM^ and analyzed by LEGENDplex^TM^ Data Analysis Software (BioLegend, San Diego, CA, USA) according to the protocols provided by the manufacturers.

### 2.6. Assessment of Anaphylaxis by Fall in Rectal Temperature

Sensitized and naïve mice that fasted for more than 2 h were challenged with OVM (20 mg/200 μL/head) using feeding needles on day 25 after the initial epicutaneous sensitization, and then changes in rectal temperature were measured 10 min before and after allergen load every 10 min up to 90–120 min using a Microprobe Thermometer BAT-12 (Physitemp, Clifton, NJ, USA). As a positive control experiment of food allergy, 1 mg OVM was also challenged to the sensitized mice by intraperitoneal route. As an oral challenge test in the cow’s milk allergy model, the fasted mice received α-casein (12 mg/200 μL/head) orally, and then changes in rectal temperature were measured.

### 2.7. Oral Administration of ASA to Amplify Mild Allergic Symptoms Induced by Oral Allergen Challenge

To amplify mild allergic reactions induced by oral allergen challenge in epicutaneous adjuvant-free allergen-sensitized mice, ASA or its derivative was administered orally as a pre-medication at the time of oral allergen challenge, mimicking ASA pre-treatment used in food-dependent exercise-induced anaphylaxis in humans [17,18]. Fasted mice were given 100 μL of ASA in 50% EtOH, L-ASA in 5% EtOH, or 50% EtOH orally before, after or at the time of allergen challenge, and then changes in rectal temperature (delta T: ΔT) were monitored. The results were compared to those without ASA treatment. To determine the most effective ASA dose, ASA was administered at 10 to 300 mg/kg at 30 min before allergen challenge. To determine the best timing of ASA treatment, the sub-maximal dose of ASA (50 mg/kg) was applied 60, 30, 15, 0 min before or 15 min after OVM challenge, and the ΔT and symptom score were monitored.

### 2.8. Assessment of Allergic Hypersensitivity Reactions Using a Modified Symptom Scoring System

To evaluate the symptoms and signs of systemic and local allergic symptoms—other than changes in rectal temperature after oral treatment with 50 mg/kg ASA for OVM allergy model mice and 100 mg/kg ASA for casein allergy model mice at 30 min before oral allergen challenge—allergic symptoms were monitored over a period of 90 min using the symptom scoring system reported previously [11,12,13]: 0 = no symptoms; 1 = scratching and rubbing around the nose and head; 2 = puffiness around the eyes and mouth, diarrhea, pilar erecti, reduced activity, and/or decreased activity with increased respiratory rate; 3 = wheezing, labored respiration, and cyanosis around the mouth and the tail; 4 = no activity after prodding or tremor and convulsion; 5 = death. The modified symptom score manifestations between the different treatment groups were compared not only by using the maximum scores but also by the frequency of recorded score 1 (local symptoms) and that of score 3 (systemic symptoms) in each group.

### 2.9. Statistical Analysis

Statistical analysis was performed using JMP software, version 14.2.0 (SAS Institute Inc, Cary, NC, USA). Data are expressed as mean ± SEMs. Statistical significance was analyzed by Wilcoxon rank sum test or Tukey-Kramer methods. Significance for all statistical tests was set at *p* < 0.05 (*) and *p* < 0.01 (**).

## 3. Results

### 3.1. Epicutaneous OVM Sensitization Induces Severe Hypersensitivity Reactions by Intraperitoneal OVM Challenge but Not by Oral Challenge

**Figure 1:**
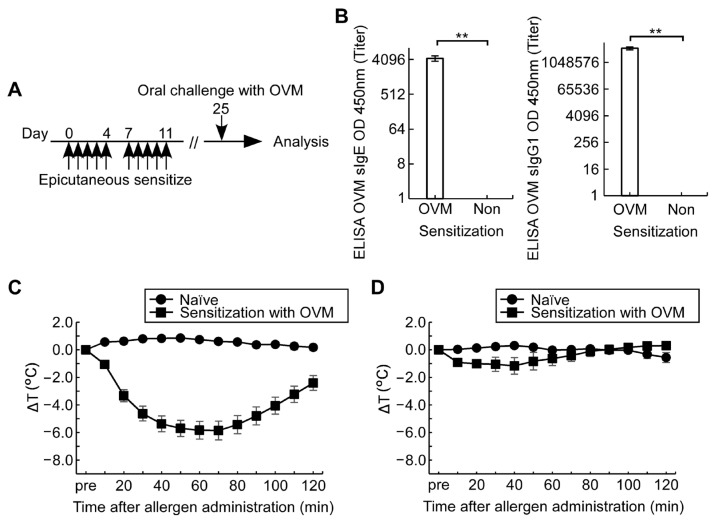
Experimental design. For epicutaneous sensitization, the shaved back skin of BALB/c female mice (*n* = 5–10/group) was treated with 4% SDS and 1 mg OVM, without adjuvant, five times a week in the first 2 weeks. On day 25, mice were orally challenged with OVM and allergic reactions were monitored. (**A**) Experimental protocol; (**B**) Serum levels of OVM sIgE and sIgG1 on day 11; (**C**) ΔT after intraperitoneal challenge with 1 mg OVM; (**D**) ΔT after oral challenge with 20 mg OVM. Data are mean ± SEMs. ** *p* < 0.01.

Epicutaneous sensitization with OVM (10 times in 2-week period) resulted in the generation of OVM sIgE and sIgG1 in serum as detected on day 11 after the initial treatment (Figure 1A,B). Intraperitoneal OVM challenge (1 mg) on day 25 induced a significant fall in rectal temperature compared to naïve mice (Figure 1C). In contrast, oral OVM challenge (20 mg) in sensitized mice did not induce a significant decrease in rectal temperature (Figure 1D). These results indicate that epicutaneous exposure to OVM induces hypersensitivity states of allergic reaction, whereas the reaction evoked by natural oral allergen challenge was mild and not enough to allow proper evaluation.

### 3.2. Compound Effect of Oral ASA Administration on Allergic Hypersensitivity Reactions

**Figure 2:**
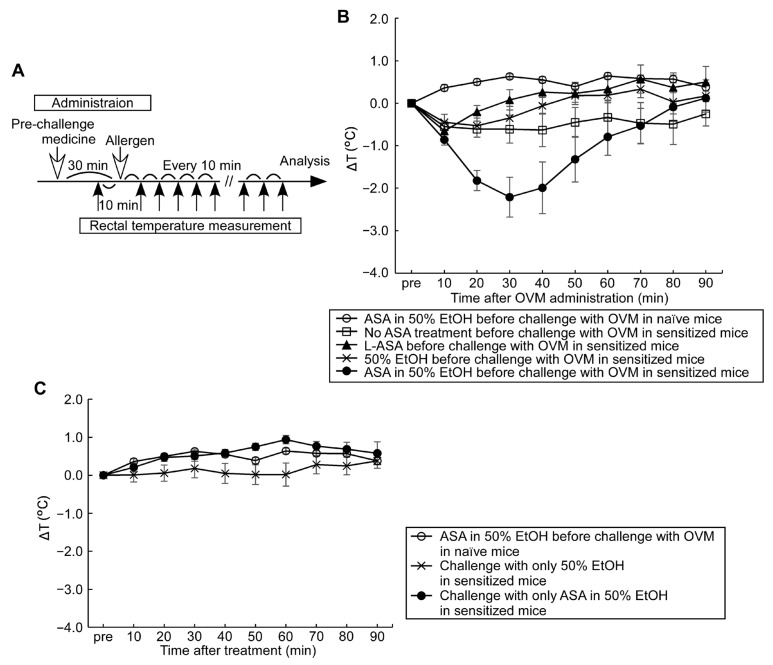
Effects of oral pre-medication with ASA and its derivative, L-ASA on oral-OVM-challenge-induced fall in rectal temperature. (**A**) Oral challenge protocol (*n* = 10/group); (**B**) ΔT after 20 mg OVM challenge in sensitized and naïve mice with and without 30 min pre-medication of ASA at 50 mg/kg, an equivalent dose of L-ASA at 91 mg/kg and 50% EtOH; (**C**) ΔT without oral OVM challenge in sensitized mice pre-medicated with ASA at 50 mg/kg or 50% EtOH and ΔT in naïve mice treated with 50 mg/kg ASA and OVM challenge as a comparison group. Data are mean ± SEMs.

Next, we explored the potential of ASA treatment in exacerbating the mild allergic hypersensitivity reactions induced by oral allergen challenge in sensitized mice. Several previous clinical studies reported that pre-medication with ASA amplified the appearance of IgE-mediated exercise-induced human wheat allergy symptoms [17,18,19] and suggested that ASA seems to increase allergen absorption from the gastrointestinal tract, with a resultant increase in circulating allergen levels in patients and animal models [19,20]. In this arm of the study, we tested the effects of two forms of aspirins; ASA in 50% EtOH, and L-ASA in 5% EtOH administered orally 30 min before allergen challenge. Oral OVM challenge alone induced a slight fall in rectal temperature in sensitized mice and almost no fall was detected in naïve mice (Figure 2B,C). Pre-medication with ASA in 50% EtOH followed by oral OVM challenge resulted in the largest drop in rectal temperature, whereas pre-medication of L-ASA in 5% EtOH and 50% EtOH as a vehicle followed by oral OVM challenge had no significant effect on rectal temperature. Other control experiments of oral OVM challenge without ASA pre-medication and ASA pre-medication alone showed no significant change in rectal temperature in the sensitized mice. These results indicate that pre-medication with ASA selectively amplified hypersensitivity allergic reactions evoked by oral OVM challenge.

**Figure 3:**
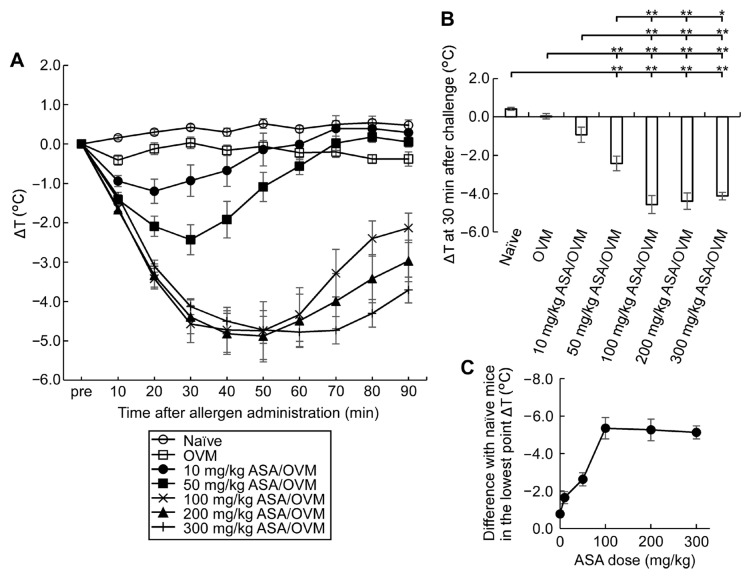
Dose-dependent effect of ASA on fall in rectal temperature after oral allergen challenge. (**A**) ΔT after 20 mg OVM challenge in allergen-sensitized and naïve mice with and without 30 min pre-medication with each ASA dose (*n* = 5–10/group); (**B**) ΔT correlations between sensitized and naïve mice with and without pre-medication of ASA; (**C**) Dose-dependent effect of pre-medication of ASA on the lowest point ΔT in sensitized mice compared with naïve mice after OVM challenge. Data are mean ± SEMs. * *p* < 0.05, ** *p* < 0.01.

To determine the optimal dose of ASA necessary to potentiate allergic reactions induced by oral allergen challenge in sensitized mice, we tested the effects of various doses of oral ASA (Figure 3). The fall in rectal temperature induced by oral OVM challenge worsened with ASA pre-medication and this effect was ASA dose-dependent from 10 to 100 mg/kg, and the lowest point ΔT reached a plateau above 100 mg/kg ASA.

**Figure 4:**
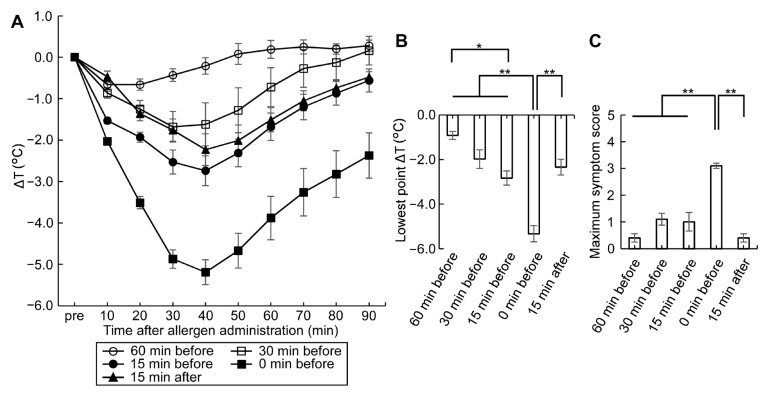
Optimal timing of ASA administration relative to oral allergen challenge. (**A**) Effects of sub-maximal dose at 50 mg/kg ASA before and after 20 mg OVM challenge on ΔT in sensitized mice; (**B**) Effects of timing of 50 mg/kg ASA medication on the lowest point ΔT in sensitized mice; (**C**) Effects of timing of 50 mg/kg ASA medication on the maximum symptom score in sensitized mice. Data are mean ± SEMs (*n* = 10/group). * *p* < 0.05, ** *p* < 0.01.

To determine the optimal timing of ASA medication, the sub-maximal dose of 50 mg/kg ASA was administered at various time points before and after oral OVM challenge. The largest fall in rectal temperature and the maximum symptom score were observed when ASA and OVM were administered simultaneously (Figure 4). The effect of ASA treatment on the lowest point ΔT was minimal at 60 min before OVM administration (Figure 4B) and the maximum symptom score was also minimal at 60 min before and 15 min after OVM administration (Figure 4C). These results indicate that the compounding effect of ASA on allergic hypersensitivity reaction is dependent on its dose as well as the time of administration relative to that of orally administered allergen. Fall in rectal temperature and the maximum symptom score showed that the optimal timing of ASA medication was oral allergen and ASA administration simultaneously or ASA administration within 15–30 min before allergen challenge.

### 3.3. ASA Amplifies Symptom Score, a Useful Tool for Monitoring Allergic Hypersensitivity Reactions

**Figure 5:**
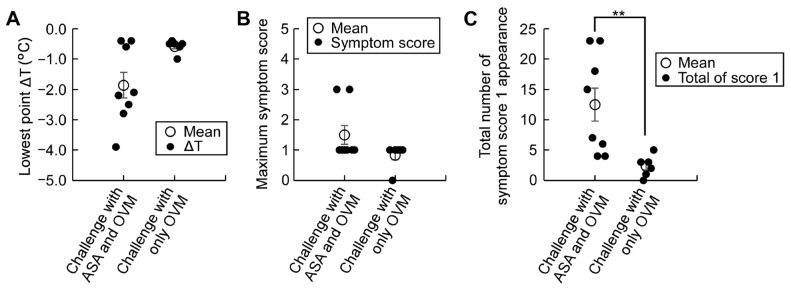
Effects of ASA pre-medication on the modified symptom score evaluation system after oral allergen challenge in sensitized mice. Allergic reactions after OVM challenge in sensitized mice were compared with and without ASA-pre-medication at 50 mg/kg. (**A**) Comparison of the effects on the lowest point ΔT; (**B**) Comparison of the effects on the maximum symptom score; (**C**) Total number of recorded symptom score 1 after allergen challenge for 90 min. Data are mean ± SEMs. (*n* = 6–8/group) ** *p* < 0.01.

In our model of epicutaneous allergen sensitization, treatment with ASA proved useful in augmentation and, thus, detection of even mild hypersensitivity reactions. Apart from changes in rectal temperature, we found that the symptom score which monitors local and systemic symptoms was useful in evaluating allergic reactions, with high sensitivity (Figure 5). The sensitivity of detection of allergic reactions by the maximum symptom score evoked by an oral 20 mg OVM challenge, with and without 50 mg/kg ASA, was almost similar to that by rectal temperature at lowest ΔT (Figure 5A,B). Furthermore, analysis of the total number of recorded local allergy symptom score 1 showed that ASA pre-medication significantly increased local allergy symptoms compared with no ASA treatment (Figure 5C). Furthermore, analysis of the frequency of recorded allergy symptom scores 1 and 3 (data shown in Supplementary Materials, Figure S1) also clearly showed the compounding effect of ASA on the local and systemic allergy symptoms.

### 3.4. High mMCPT-1 Blood Levels Induced by Combination of Oral OVM Challenge Plus ASA and Splenocyte Cytokine Productions

**Figure 6:**
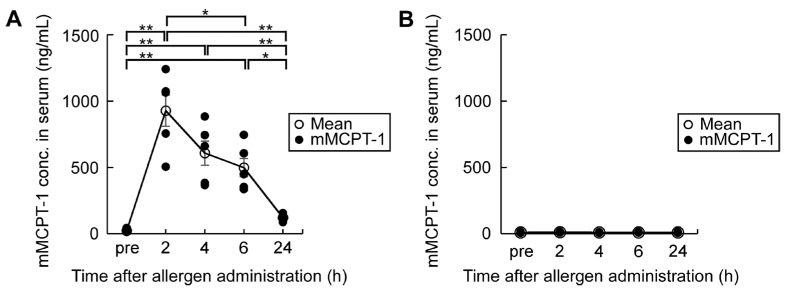
Serial changes in serum mMCPT-1 levels before and after oral administration of 20 mg OVM and ASA pre-medication at 50 mg/kg. (**A**) Changes in mMCPT-1 levels in sensitized mice (*n* = 5/group); (**B**) Changes in mMCPT-1 levels in naïve mice (*n* = 6/group). Data are mean ± SEMs. * *p* < 0.05, ** *p* < 0.01.

We analyzed the combined effects of oral OVM challenge and 50 mg/kg ASA on mMCPT-1 blood levels (Figure 6). Oral allergen challenge plus ASA resulted in an increasing trend in blood mMCPT-1 levels, relative to baseline levels measured before OVM load, with a peak value recorded at 2 h, then returning to almost the baseline level at 24 h in allergen-sensitized mice. In contrast, oral administration of OVM plus ASA in naïve mice did not show such changes in blood mMCPT-1 levels.

OVM-specific ex vivo production levels of Th1 cytokine (IFN-γ) and Th2 cytokines (IL-4, IL-5, IL-6, IL-9 and IL-13) in the culture media from splenocytes of OVM-sensitized mice and naïve mice were analyzed (Table 1). Although OVM-induced increase in Th1 cytokine (IFN-γ) levels for sensitized mice was not significantly different from the level for naïve mice, increase in the levels of all Th2 cytokines tested was significantly different. Among Th2 cytokines, prominent increases were seen in particular in IL-5, IL-9 and IL-13.

### 3.5. Establishment of an Adjuvant-Free Casein-Sensitized Cow’s Milk Allergy Mouse Model and Analysis of Allergic Hypersensitivity Reactions

**Figure 7:**
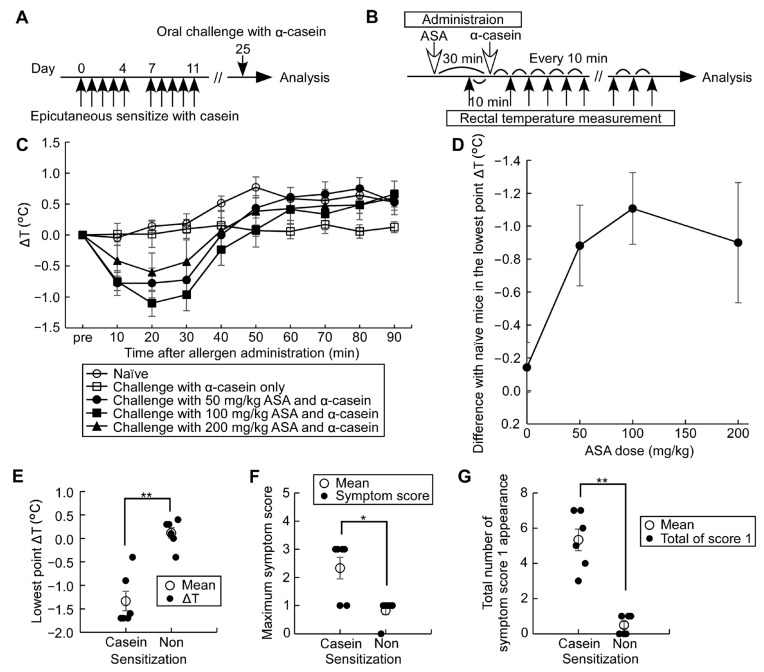
Design of adjuvant-free cow’s milk allergy experiments. (**A**) Experimental protocol; (**B**) Oral challenge protocol of 12 mg α-casein combined with 30 min ASA-pre-medication; (**C**) Serial changes in ΔT in casein-sensitized and naïve mice pre-treated with different doses of ASA (*n* = 6–8/group); (**D**) Dose-dependent effect of ASA pre-medication on the lowest point ΔT in casein-sensitized mice. The following results (**E**–**G**) were comparisons between casein-sensitized and naïve mice treated with oral α-casein challenge with ASA pre-medication at 100 mg/kg; (**E**) Comparison of the lowest point ΔT; (**F**) Comparison of the maximum symptom score; (**G**) Comparison of total number of recorded symptom score 1 after allergen challenge for 90 min. Data are mean ± SEMs. * *p* < 0.05, ** *p* < 0.01.

The major allergen of cow’s milk is casein, accounting for 80% of total milk proteins and being highly antigenic, making it responsible for the strongest systemic allergic reactions [21]. Furthermore, α-casein is a water-soluble dominant component of casein and not found in human milk. Since there have been no previous reports of adjuvant-free casein-sensitized allergy mouse models, in the second arm of the study, we established a cow’s milk allergy mouse model using cow’s milk casein without adjuvants. Mice were epicutaneously sensitized with 1 mg of casein, using the same method described above for OVM sensitization. Allergic hypersensitivity reactions were induced by oral challenge with 12 mg α-casein, together with 100 mg/kg ASA administered 30 min before the allergen challenge. The change in rectal temperature (ΔT) over a period of 90 min was used to assess the severity of allergic reactions (Figure 7A–D). The lowest point ΔT varied according to the dose of ASA with the maximum change noticed at an ASA dose of 100 mg/kg. The allergic hypersensitivity reactions induced by the above protocol were also assessed by the modified symptom score by monitoring maximum symptom score, total number of recorded symptom score 1, frequency of recorded symptom score 1, and that of score 3 (data not shown), in addition to ΔT (Figure 7E–G). In the casein-sensitized mouse group, but not the naïve group, pre-medication with ASA clearly showed the allergic reactions induced by oral α-casein challenge.

### 3.6. Casein Immunotherapy Monitoring

**Figure 8:**
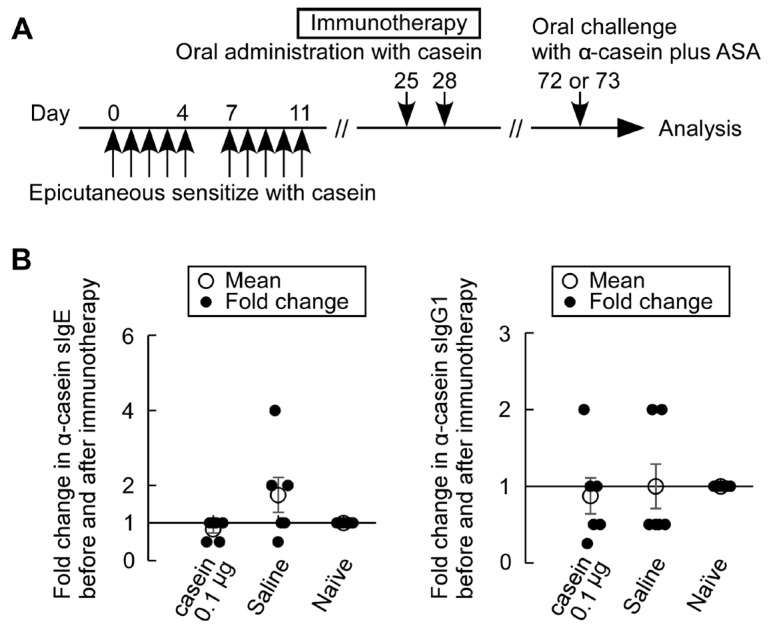
Changes in α-casein sIgE and sIgG1 levels by oral casein immunotherapy in adjuvant-free casein-sensitized allergy model mice. (**A**) Experimental protocol. As an oral immunotherapy, 0.1 µg/head of casein was administered orally two times on day 25 and 28 in sensitized mice. As a control, saline was administered orally. On day 72 or 73, mice were challenged with ASA and α-casein, then rectal temperature and symptom score were monitored (*n* = 6/group); (**B**) Fold changes in serum α-casein sIgE and sIgG1 levels after immunotherapy of casein or saline in the sensitized and naïve mice. Serum samples were collected before immunotherapy on day 24 and, after, on day 64 (*n* = 6/group).

Oral casein immunotherapy was conducted using an adjuvant-free casein-sensitized allergy mouse model described above. As an initial safety trial, we attempted to administer a minimal oral dose of casein immunotherapy at 0.1 μg/head and limited number of treatments on days 25 and 28 to avoid severe adverse reactions (Figure 8). Fold changes in the levels of α-casein sIgE in serum before and after immunotherapy showed that suppression tendency, though not significant, of fold increases in α-casein sIgE levels in casein immunotherapy mice compared with saline-treated mice. No trends of change in sIgG1 levels in immunotherapy-treated mice and α-casein sIgE and sIgG1 levels in naïve mice were observed.

**Figure 9:**
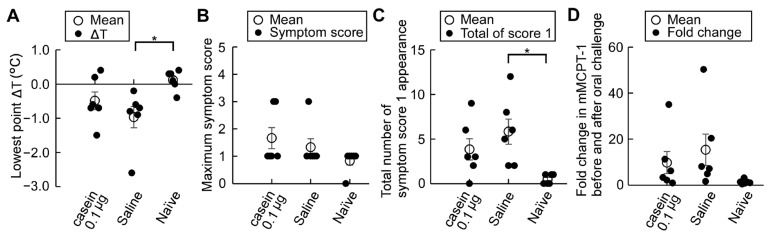
Effects of allergic symptoms after casein immunotherapy in adjuvant-free casein-sensitized allergy model mice. (**A**) Comparison of the lowest point ΔT and (**B**) the maximum symptom score after oral α-casein challenge with ASA pre-medication in casein- and saline-treated sensitized and naïve mice; (**C**) Total number of recorded symptom score 1 after oral α-casein challenge in casein- and saline-treated sensitized and naïve mice; (**D**) Fold changes in serum mMCTP-1 levels after oral α-casein challenge. Serum samples were collected before on day 64 and after α-casein challenge on day 72 or 73 (*n* = 6/group). Data are mean ± SEMs. * *p* < 0.05.

We then evaluated allergic reactions in these mice after oral challenge with 12 mg α-casein, together with 100 mg/kg ASA, on day 72 or 73 (Figure 9). Mild suppression tendencies of the lowest point rectal temperature (ΔT), the total number of symptom score 1 and fold changes in mMCPT-1 levels before and after oral challenge in mice treated with casein immunotherapy, though not significant, were observed compared in mice treated with saline (Figure 9A,C,D). In addition, no suppression trends of the maximum symptom score (Figure 9B) and the frequency of symptom score 3 (data not shown) in mice treated with casein immunotherapy were observed compared with saline-treated mice.

## 4. Discussion

In the present study, we developed two mouse models of food allergy by epicutaneous sensitization with chicken egg OVM and cow’s milk casein, without the use of any adjuvant, such as cholera toxin, alum and Freund’s adjuvant [11,21,22,23], followed by oral, rather than intravenous or intraperitoneal, allergen administration to evoke allergic reactions. In addition, we established a simple and sensitive method to amplify allergic symptoms in mild cases.

For epicutaneous allergen sensitization, the back skin was shaved then treated with 4% SDS. This compound is known to damage the tight junction barriers in the epithelium [14,15]. Topical application of SDS is used as an alternative for impairing the skin barrier to atopic dermatitis or other skin inflammatory conditions in the pathogenesis of human food allergy. SDS and related reagents are commonly present in many commercial products, such as detergents, soaps and shampoos, and traces of these detergents in washed towels or clothes, even at 100,000-fold dilution (vol/vol), are known to decrease tight junction barrier integrity in human keratinocytes [14]. Importantly, the recently proposed “epithelial barrier hypothesis” states that damage to the epithelial barrier by external factors is followed by entry of bacteria and allergens, which can subsequently induce chronic inflammation [24]. In the present study, the efficacy of SDS/epicutaneous allergen sensitization in the development of food allergy was analyzed by detection of sIgE and sIgG1 antibodies in serum, induction of anaphylaxis by oral allergen challenge, and increase in blood levels of mMCPT-1, a marker of activation of mast cells (Figure 1, Figure 6 and Figure 7). In addition, the profile of prominent increase in the Th2 cytokines secreted from the splenocytes of OVM-sensitized mice (Table 1) was consistent with cytokine patterns in allergic mice reported [25,26]. These results indicate that OVM and casein allergy mouse models could be created successfully without the use of adjuvants followed by oral administration of allergens, mimicking the case in humans. To our knowledge, this is the first report that describes chicken egg OVM and cow’s milk casein allergy mouse models induced by epicutaneous adjuvant-free allergen sensitization followed by oral allergen challenge with ASA to amplify mild allergic symptoms.

To evaluate the mild food allergic symptoms in our model, we opted to use methods applied in humans that are known to amplify symptoms, rather than increasing the dose of allergen administration or apply the allergen intraperitoneally or intravascularly. Oral administration of ASA is a promising tool for allergic symptom amplification and is reported to augment food-dependent exercise-induced anaphylaxis in humans [17]. Whereas the initial use of ASA for this purpose was coupled with exercise, subsequent research indicated that ASA alone can augment symptoms of food-dependent anaphylaxis [18]. In the present study, we determined the optimal dose of ASA and the best time to administer ASA in relation to food allergen challenge (Figure 2, Figure 3 and Figure 4). ASA, which is insoluble in water, was dissolved in 50% EtOH, while L-ASA derivative, which has increased hydrophilicity, was dissolved in 5% EtOH, and their effects on allergic symptom amplification were examined. Ingested ASA and L-ASA equally act as inhibitors of the lipoxygenase and the cyclooxygenase pathways and have been used for the diagnosis of aspirin-sensitive asthma [27,28]. As shown in Figure 3, the fall in rectal temperature (ΔT) worsened significantly with ASA in a dose-dependent manner up to 100 mg/kg of body weight. However, no significant potentiation of ΔT was detected with L-ASA and 50% EtOH (Figure 2). With regard to the timing of ASA, Figure 4 shows that ΔT was efficiently enhanced when ASA and food allergens were orally administered simultaneously or within 30 min before and 15 min after allergen challenge. The effect of ASA was greatly diminished when it was administered at 60 min before allergen challenge. Although the best response was observed when ASA and allergen were administered simultaneously, ASA was applied in our experiments at 30 min before allergen administration. The reasons for this approach are related to the solvents used with ASA and allergens, which are sometimes different and cannot be mixed with each other, as well as to the difficulty in simultaneous administration on a large number of animals.

The above data suggest that the symptom exacerbation effects of ASA are not due to inhibition of lipoxygenase and cyclooxygenase pathways, which is considered to play a role in ASA- and L-ASA-induced asthma in humans [27,29,30]. The different effects noticed in our study between ASA and L-ASA may be related to the known stimulatory effect of ASA on the gastrointestinal mucosa, particularly on the gastric mucosa [31,32], compared with the milder gastrotoxicity of L-ASA [33]. ASA is also known to enhance intestinal ovalbumin absorption in rodents by inducing intestinal barrier disruption and increasing paracellular permeability [20,34]. That the strongest hypersensitivity and clearest symptoms were noted with simultaneous administration of allergen and ASA (Figure 4) strongly suggests that ASA acts in our model as a transitory irritant of the mucosal lining of gastrointestinal tract.

In the present study, we also improved the standard allergic symptom evaluation system used to assess the severity of allergic hypersensitivity [11,12,13]. Specifically, while the conventional symptom score evaluates the highest value for each symptom appearing during the observation period, the system used in our study evaluated not only the maximum score but also the frequency of each symptom score. This was made possible by observing the allergic symptoms at every 10 min interval over the entire period of 90 min, together with rectal temperature recording (Figure 5 and Figure 7E–G, Supplementary Figure S1). Our system provides more detailed evaluations on even mild allergic symptoms.

Recently, oral immunotherapy has been demonstrated to be an efficient option for the management of food allergy. Cow’s milk allergy is one of the most prevalent food allergies in young children in many countries. In patients with cow’s milk allergy, the efficacy of oral immunotherapy was reported to be 40% to 90%, with a significant increase in the threshold intake [35,36,37,38,39,40], but it can induce serious adverse reactions such as anaphylaxis [41]. In order to develop a safer and more effective immunotherapy for IgE-mediated cow’s milk allergy, we are attempting to use the above mouse model we have developed. Humoral changes caused by oral immunotherapy in humans include a decrease in sIgE levels and a rise in sIgG levels, especially IgG4. Our casein immunotherapy with a minimal dose of casein at 0.1 μg/head and two treatment times showed suppression tendency for an increase in α-casein sIgE levels with no change in sIgG1 levels (Figure 8). With the suppression tendency, there were reduction trends observed, though not of significance, in the lowest point ΔT, the total number of symptom score 1 appearances, and fold changes in mMCTP-1 levels (Figure 9). However, a minimal dose of casein immunotherapy at 0.1 μg/head was not enough to suppress systemic allergic reactions (symptom score 3, data not shown) and the maximum symptom score values. Further studies are needed to find more effective and safer cow’s milk allergy immunotherapy.

The immunological mechanisms of thymic stromal lymphopoietin (TSLP)–basophil axis mediated food allergy triggered by epicutaneous food allergen sensitization have been reported precisely [42], with an increased serum IgE and Th2 cytokine responses and mast cells in the small intestine associated with allergic symptoms. We also detected an increase in IL-4 and IL-5 mRNA expressions in mesenteric lymph nodes early after epicutaneous OVM sensitization (data not shown). In many animal model studies, exogenous adjuvants and/or reagents, such as vitamin D analog (calcipotriol), that activate immune systems had been used for effective transdermal antigen sensitization. The aim of our study was to develop an animal model for the analysis of pathogenesis and treatment of a large number of mild to moderate allergic patients, other than those with severe allergies. The allergens used were adjuvant-free OVM and casein, which have no inherent adjuvant activity like peanut allergen reported [43], and induced food allergies with a milder transdermal sensitization than the mechanically disrupted skin barrier by the tape-stripped method [25,42]. This study established a mouse model that mimics the onset of human food allergy, and also clarified the optimal treatment timings and doses of ASA used as a sensitizer that allows valuation of even mild allergic symptoms, enabling future development and evaluation of treatment, such as food allergen immunotherapy.

Our study has certain limitations. First, we proposed an improved allergic symptom evaluation system to evaluate the severity of allergic symptoms in mice treated with ASA in addition to monitoring changes in rectal temperature. However, this method is partly subjective. Thus, we are currently engaged in the design of a more robust method for evaluation of symptom score that relies on a video recording evaluation system. Second, the food allergy models described in this study involved epicutaneous allergen sensitization. However, food allergy may also be caused by oral or respiratory sensitization other than epicutaneous sensitization, depending on the type of allergens. Therefore, we are also currently engaged in establishing allergen sensitization models that are based on different routes other than epicutaneous sensitization. We expect the results of these new studies to be available in the near future.

In conclusion, we developed two mouse allergy models of chicken egg OVM and cow’s milk casein, each induced by epicutaneous allergen sensitization followed by oral allergen challenge. Because allergic symptoms in the adjuvant-free model were mild in nature, they were amplified by pre-medication with ASA. In addition, the severity of allergic symptoms was evaluated by both rectal temperature recording and a sensitive reformulated symptom score system. We are confident that employment of our new animal models in future studies mimicking pathogenesis of food allergy in humans will be helpful in enhancing our understanding of food allergy and the design of new treatments and prevention methods.

## Figures and Tables

**Table 1:** Cytokine levels in culture media of splenocytes stimulated with (+) or without (−) OVM.

Cytokine ^1^	OVM Stimulation	OVM-Sensitized Mice (*n* = 6)	Naïve Mice (*n* = 6)
IFN-γ	−	1286.2 ± 337.5	2197.7 ± 1217.5
	+	4818.3 ± 1023.4	1570.5 ± 919.3
IL-4	−	17.3 ± 3.2	11.0 ± 2.5
	+	26.0 ± 1.8 **	7.5 ± 2.7
IL-5	−	7.2 ± 2.2	1.0 ± 0.6
	+	378.5 ± 82.1 **	1.8 ± 1.1
IL-6	−	147.2 ± 28.4	72.4 ± 25.8
	+	759.5 ± 133.0 **	58.5 ± 22.1
IL-9	−	74.9 ± 14.8	54.4 ± 28.0
	+	800.8 ± 135.5 **	50.5 ± 26.6
IL-13	−	33.6 ± 5.1	26.7 ± 11.2
	+	314.8 ± 55.1 **	18.5 ± 8.9

^1^ Data (pg/mL) are mean ± SEMs. ** *p* < 0.01, significant difference of cytokine levels for OVM-sensitized mice vs. naïve mice.

## Data Availability

The data presented in this study are available in insert article and supplementary material here.

## Supplementary Materials

The following are available online at: https://www.mdpi.com/article/10.3390/nu15030757/s1, Figure S1: Effects of ASA pre-medication on the modified symptom scores 1 and 3.
