# Supplementary material for: A Murine Model of Food Allergy by Epicutaneous Adjuvant-Free Allergen Sensitization Followed by Oral Allergen Challenge Combined with Aspirin for Enhanced Detection of Hypersensitivity Manifestations and Immunotherapy Monitoring
Source: Nutrients. 2023 Feb 2;15(3):757. doi: 10.3390/nu15030757 (PMC9920581; doi:10.3390/nu15030757)
Supplement: Supplementary file 1 [file nutrients-15-00757-s001.zip › nutrients-2153071-supplementary.pdf]

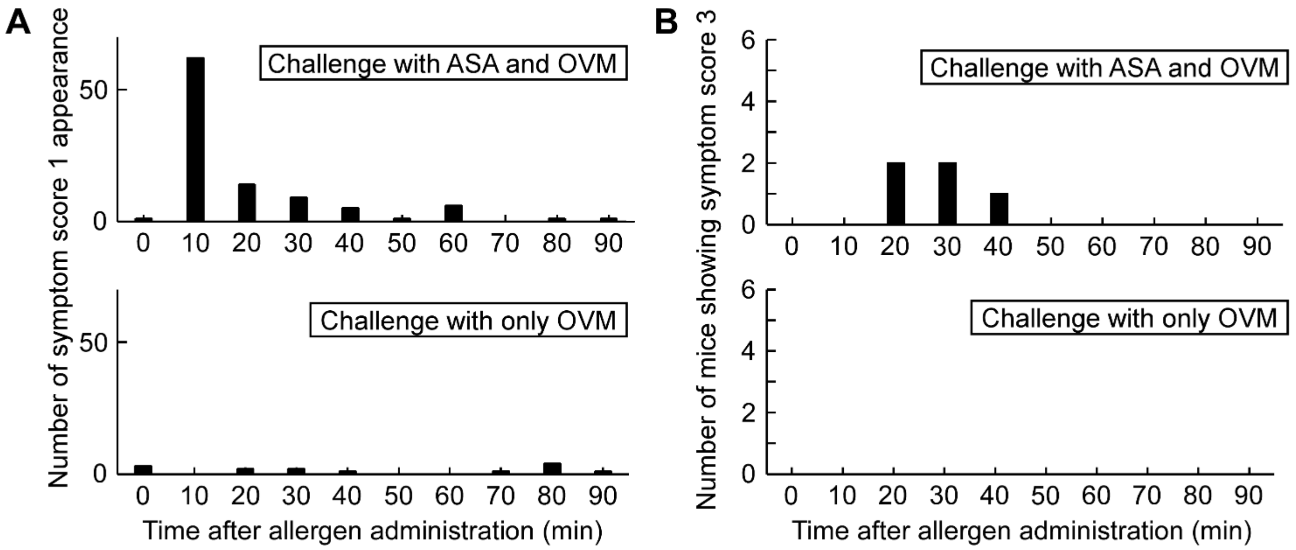

Figure S1. Effects of ASA pre-medication on the modified symptom scores 1 and 3.
(A) Number of recorded symptom score 1; **(B)** Number of recorded symptom score 3 at each time point during the 90-min monitoring period after oral OVM challenge combined with and without 30-min-ASA-pre-medication at 50 mg/kg in OVM sensitized mice (detailed data for Figure 5). (*n* = 6–8/group)
